# Differential Effects of Dietary Macronutrients on the Development of Oncogenic KRAS-Mediated Pancreatic Ductal Adenocarcinoma

**DOI:** 10.3390/cancers14112723

**Published:** 2022-05-31

**Authors:** Liang Zhu, Juntao Ji, Jianjia Ma, Dan Wang, Muyun Liu, James Xianxing Du, Rong Chen, Wei Hou, James L. Abbruzzese, Craig D. Logsdon, Vincent W. Yang, Yongde Luo, Weiqin Lu

**Affiliations:** 1 Department of Medicine, Stony Brook University, Stony Brook, NY 11794, USA; dr_zhuliang@163.com (L.Z.); ji-jt@163.com (J.J.); jianjia.ma@stonybrookmedicine.edu (J.M.); jsjdwangdan08@163.com (D.W.); james.du@stonybrookmedicine.edu (J.X.D.); vincent.yang@stonybrookmedicine.edu (V.W.Y.); yongdeluo08@wmu.edu.cn (Y.L.); 2 The First Affiliated Hospital, School of Pharmacological Sciences, Wenzhou Medical University, Wenzhou 325015, China; 3 Department of Cancer Biology, University of Texas MD Anderson Cancer Center, Houston, TX 77030, USA; xhliumuyun@163.com (M.L.); clogsdon@mdanderson.org (C.D.L.); 4 Department of Experimental Therapeutics, University of Texas MD Anderson Cancer Center, Houston, TX 77030, USA; rchen@mdanderson.org; 5 Department of Family, Population & Preventive Medicine, Stony Brook University, Stony Brook, NY 11794, USA; wei.hou@stonybrookmedicine.edu; 6 Division of Medical Oncology, Department of Medicine, Duke Cancer Institute, Duke University, Durham, NC 27710, USA; james.abbruzzese@duke.edu

**Keywords:** pancreatic cancer, KRAS, macronutrients, high-carbohydrate diet, inflammation, high-protein diet

## Abstract

**Simple Summary:**

Pancreatic ductal adenocarcinoma (PDAC) is a highly lethal disease and oncogenic KRAS mutations are prevalent in PDAC patients. Oncogenic KRAS is critical for the initiation and development of PDAC; however, it alone is insufficient to drive pancreatic cancer. Oncogenic KRAS is an important mediator in metabolic reprogramming, including glucose, glutamine, and fatty acid metabolisms, for cancer cell survival, proliferation, and invasion. Oncogenic KRAS and chronic high-fat diet synergize to promote PDAC, suggesting that dietary intake is critically involved in PDAC development. Here, by feeding mice expressing an endogenous level of oncogenic KRAS^G12D^ with a high-fat diet, high-carbohydrate diet, high-protein diet, or normal diet, we have demonstrated that different dietary macronutrients differentially impact pancreatic cancer susceptibility, and a high-protein diet may represent the desired, favorable dietary choice compared to high-fat diet and high-carbohydrate diet for pancreatic cancer patients. In addition, management of macronutrient intake aimed at thwarting inflammation is a promising preventive strategy for patients harboring oncogenic KRAS.

**Abstract:**

KRAS mutations are prevalent in patients with pancreatic ductal adenocarcinoma (PDAC) and are critical to fostering tumor growth in part by aberrantly rewiring glucose, amino acid, and lipid metabolism. Obesity is a modifiable risk factor for pancreatic cancer. Corroborating this epidemiological observation, mice harboring mutant KRAS are highly vulnerable to obesogenic high-fat diet (HFD) challenges leading to the development of PDAC with high penetrance. However, the contributions of other macronutrient diets, such as diets rich in carbohydrates that are regarded as a more direct source to fuel glycolysis for cancer cell survival and proliferation than HFD, to pancreatic tumorigenesis remain unclear. In this study, we compared the differential effects of a high-carbohydrate diet (HCD), an HFD, and a high-protein diet (HPD) in PDAC development using a mouse model expressing an endogenous level of mutant KRAS^G12D^ specifically in pancreatic acinar cells. Our study showed that although with a lower tumorigenic capacity than chronic HFD, chronic HCD promoted acinar-to-ductal metaplasia (ADM) and pancreatic intraepithelial neoplasia (PanIN) lesions with increased inflammation, fibrosis, and cell proliferation compared to the normal diet (ND) in *Kras^G12D/+^* mice. By contrast, chronic HPD showed no significant adverse effects compared to the ND. Furthermore, ablation of pancreatic acinar cell cyclooxygenase 2 (*Cox-2*) in *Kras^G12D/+^* mice abrogated the adverse effects induced by HCD, suggesting that diet-induced pancreatic inflammation is critical for promoting oncogenic KRAS-mediated neoplasia. These results indicate that diets rich in different macronutrients have differential effects on pancreatic tumorigenesis in which the ensuing inflammation exacerbates the process. Management of macronutrient intake aimed at thwarting inflammation is thus an important preventive strategy for patients harboring oncogenic KRAS.

## 1. Introduction

Pancreatic cancer is a highly lethal disease with a dismal trajectory of becoming the second leading cause of cancer-related deaths by approximately 2026 in the United States [1]. The development of PDAC, the predominant form of pancreatic cancer, is a multifactorial process in which both genetic and environmental risk factors are at play [2,3]. KRAS is a small GTPase that acts as a binary molecular switch in response to activated upstream signaling receptors while activating a plethora of signaling pathways, most notably Raf/MEK/ERK, PI3K/AKT, RalGDS/p38 MAPK, Rac and Rho, to control an array of biological processes, including survival, proliferation, migration, and metabolic adaptation [4,5,6]. Oncogenic KRAS mutations, mainly G12D and G12V, are present in as high as 95% of pancreatic cancer patients and are critical for pancreatic cancer initiation and development; however, mutant KRAS alone is insufficient to drive PDAC in adults [7,8,9,10].

Studies have revealed that in addition to genetic change, which sets the stage for the initiation and progression of PDAC, certain environmental risk factors are required as the so-called secondary hit to promote the rapid development of invasive PDAC [7,11,12,13]. The activation of mutant KRAS was shown to foster tumor growth by rewiring cancer cell metabolic pathways, which was initially revealed by its ability to promote glycolysis [14,15]. Using both in vitro and in vivo models with the tetracycline-controlled expression of mutant KRAS, studies have shown that oncogenic KRAS promoted glucose uptake, pentose phosphate pathway (PPP), hexosamine biosynthesis pathway, and the metabolic switch from oxidative phosphorylation to glycolysis, in order to provide building blocks for cancer cell survival and proliferation [16,17]. Indeed, increased expression of GLUT1 is often detected in oncogenic KRAS-induced pancreatic neoplastic lesions [18]. On the contrary, a low glycemic diet could impair tumor growth by altering cellular lipid composition [19]. The rewiring of amino acid metabolism was also observed to contribute to pancreatic cancer cell proliferation, invasion, metastasis, and redox alterations [20]. RAS-driven cancers are addicted to certain amino acids, such as glutamine, aspartate, serine, and glycine, to fuel the anabolic process and to adapt to oxidative stress [21,22,23]. It was shown that in PDAC cells, oncogenic KRAS promotes a non-canonic glutamine catabolic pathway to elevate the production of aspartate via mitochondrial aspartate transaminase, cytosolic aminotransferase, malate dehydrogenase 1, and malic enzyme 1, which increase pyruvate and NADPH critical to maintaining redox balance and PDAC growth [23]. Additionally, oncogenic KRAS-induced NRF2 could upregulate the expression of glutaminase-1, glutamate oxaloacetate transaminase 1, and cystine/glutamate antiporter SLC7A11 to enhance glutaminolysis and antioxidant capacity, leading to chemoresistance in KRAS-driven pancreatic cancer [24]. Oncogenic KRAS was also reported to downregulate hormone-sensitive lipase (HSL) for lipid droplet formation, which is critical to fuel pancreatic cancer cells during metastasis and invasion [25]. Obesity is a notable modifiable risk factor for pancreatic cancer and studies have shown that by serving as a second hit, obesogenic HFD drastically promoted oncogenic KRAS-mediated PDAC development, suggesting that pancreatic acinar cells harboring oncogenic KRAS are vulnerable to HFD challenge [12,26]. These studies suggest that dietary choices may have a critical impact on the susceptibility to neoplastic transformation.

As cancer cells harboring oncogenic KRAS show dysregulated metabolism in glucose, fats, and amino acids, it is important to understand the dietary effects on oncogenic KRAS-mediated pancreatic tumorigenesis for better patient management [16,17,27,28]. In this study, we investigated comparatively the differential effects of HFD, HCD, and HPD on oncogenic KRAS-mediated pancreatic neoplasia. Our studies showed that HCD, but not HPD, promoted PDAC development in association with oncogenic KRAS, albeit with a lesser potency than the obesogenic HFD. Ablation of COX-2 in pancreatic acinar cells suppressed HCD and oncogenic KRAS mediated inflammation, fibrosis, and PanIN lesions, suggesting a critical role of diet-induced acinar cell inflammation in the development of pancreatic neoplasia. Thus, our studies provide new insights into the role of dietary factors and new preventive strategies for pancreatic cancer.

## 2. Materials and Methods

### 2.1. Genetically Engineered Transgenic Mice

Kras^LSL-G12D/+^ mice, which carry conditional knock-in of Kras^G12D^ on one allele, were obtained as previously described [29]. Kras^LSL-G12D/+^ mice were crossed with the previously described fElas^CreERT^ mice to generate fElas^CreERT^;Kras^LSL-G12D/+^ double-transgenic mice, which express Kras^G12D^ from one allele specifically in pancreatic acinar cells upon CreERT-LoxP-mediated recombination under tamoxifen induction [30,31,32]. The double transgenic mice were further crossed with Cox-2^flox/flox^ conditional knockout mice to generate the triple transgenic mice *fElas^CreERT^*; *Kras^G12D/+^; Cox-2^−/−^* in pancreatic acinar cells as previously described [32]. All mouse lines are in C57BL/6J background. All animal experiments were reviewed and approved by The University of Texas MD Anderson Cancer Center and Stony Brook University Institutional Animal Care and Use Committee (IACUC) (Protocol #1576715 and #1603064).

### 2.2. Diets

The normal rodent chow diet (ND) (PicoLab Rodent Diet 20 5053, 0007688, LabDiet), contains 24.5% calories from protein, 13.1% from fat, and 62.4% from carbohydrates. HFD (DIO Rodent Purified Diet Blue 58Y1, 56833, Test Diet) provides 60.9% energy from fat, 18.3% from protein, and 20.1% from carbohydrates and includes 31.66% lard, 3.231% soybean oil, 4.7% linoleic acid, 0.39% linolenic acid, 25.845% casein, 8.891% sucrose, and 6.461% cellulose. The high carbohydrate diet (HCD) (DIO Rodent Purified Diet Yellow 58Y2, 56834, Test Diet) has 71.8% energy from carbohydrates, 10.2% from fat, and 18% from protein and includes 33.129% sucrose, 29.856% dextrin, and 18.956% casein. The high protein diet (HPD) (D15121605, Research Diets) has 60% energy from protein, 30% from carbohydrates, and 10% from fat, with 2400 kcalories from casein, 600 kcalories from maltodextrin, 10, 576 kcalories from corn starch, 225 kcalories from soybean oil, and 180 kcalories from lard.

### 2.3. Treatments of Mice

At 75 days of age, mice were given tamoxifen orally for 5 days to induce an endogenous level of *Kras^G12D/+^* expression specifically in pancreatic acinar cells. The mice were separated into four groups that fed an ND, an HFD, an HCD, and an HPD, respectively. The mice were either euthanized after intake of various diets for 10 weeks for the analyses of the changes in the pancreas or further maintained to record the survival curve. The body weight was measured weekly.

### 2.4. Histology

The pancreata of the mice were dissected and fixed in 10% formalin solution overnight. After incubation, formalin-fixed tissue samples were placed in cassettes, dehydrated in alcohol gradients, embedded in paraffin, and sectioned. The 5 µm-thick tissue sections were dewaxed in xylene, rehydrated through reversed ethanol gradients, washed in PBS thoroughly, and then stained with hematoxylin and eosin (H&E).

### 2.5. Immunohistochemistry (IHC)

Paraffin-embedded pancreatic tissue slides were deparaffinized by two washes with xylene, 100% ethanol, 95% ethanol, and one wash of 80% ethanol. Antigen retrieval was performed using 1× DAKO target retrieval solution (DAKO) in a steamer for 20 min at 98 °C followed by blocking endogenous peroxidases with 0.3% H_2_O_2_. Slides were then blocked using 8% fish gelatin to prevent nonspecific binding and then incubated with primary antibodies against α smooth muscle actin (α-SMA) (ab5694, 1:1000, Abcam, Cambridge, UK), amylase (sc46657, 1:500, Santa Cruz, Dallas, TX, USA), CK19 (sc33111, 1:500, Santa Cruz), Muc5 (45M1, 1:400, Invitrogen, Carlsbad, CA, USA), F4/80 (14-4801-85, 1:100, eBioscience, San Diego, CA, USA), CD3 (sc1127, 1:400, Santa Cruz), Mist1 (14896, 1:500, Cell Signaling, Danvers, MA, USA), Ki67 (RM-9106-S1, 1:300, Thermo Scientific, Waltham, MA, USA) or Cox-2 (RM-9121-S, 1:600, Thermo Scientific) overnight at 4 °C. After washing, the slides were incubated with appropriate biotinylated secondary antibodies (Vector Laboratories, Burlingame, CA, USA) at room temperature for 1 h, washed again in PBS, incubated with ABC reagent (Vector Laboratories, Burlingame, CA, USA) for 30 min, and then reacted with diaminobenzidine (DAB, Vector Laboratories, Burlingame, CA, USA) for about 3 min. The resulting slides were then counterstained with hematoxylin until the desired stain intensity developed. Fiji ImageJ software and GraphPad Prism were used to obtain data from IHC images for quantification and statistical analyses.

### 2.6. Sirius Red Staining

Pancreatic tissues were embedded in paraffin, sectioned, deparaffinized, hydrated in water, and incubated in Picro-Sirius Red solution (Abcam, ab150681) for 1 h to stain collagens. After rinsing twice in 0.5% acetic acid solution, the stained sections were dehydrated twice in alcohol and mounted. Fiji ImageJ software and GraphPad Prism were used for quantification and statistical analysis.

### 2.7. Ki67 Proliferation Index

The Ki67 proliferation index was determined as the percentage of the positively stained nuclei of ductal cells. Random fields were chosen per tissue section and positive nuclei were counted manually as described [30].

### 2.8. Statistical Analysis

Data were analyzed and graphed with Fiji ImageJ and GraphPad Prism 7, respectively. Comparison between two groups was analyzed by Student *t*-test. Differences between survival curves were assessed by performing a log-rank (Mantel-Cox) test. The body weight data were analyzed by 2-way ANOVA Tukey multiple-comparisons test. *p*-values less than 0.05 were considered statistically significant. Results are expressed as mean ± SEM.

## 3. Results

### 3.1. A High Carbohydrate Diet Promotes KRAS^G12D^-Mediated Pancreatic Neoplasia

**Figure 1:**
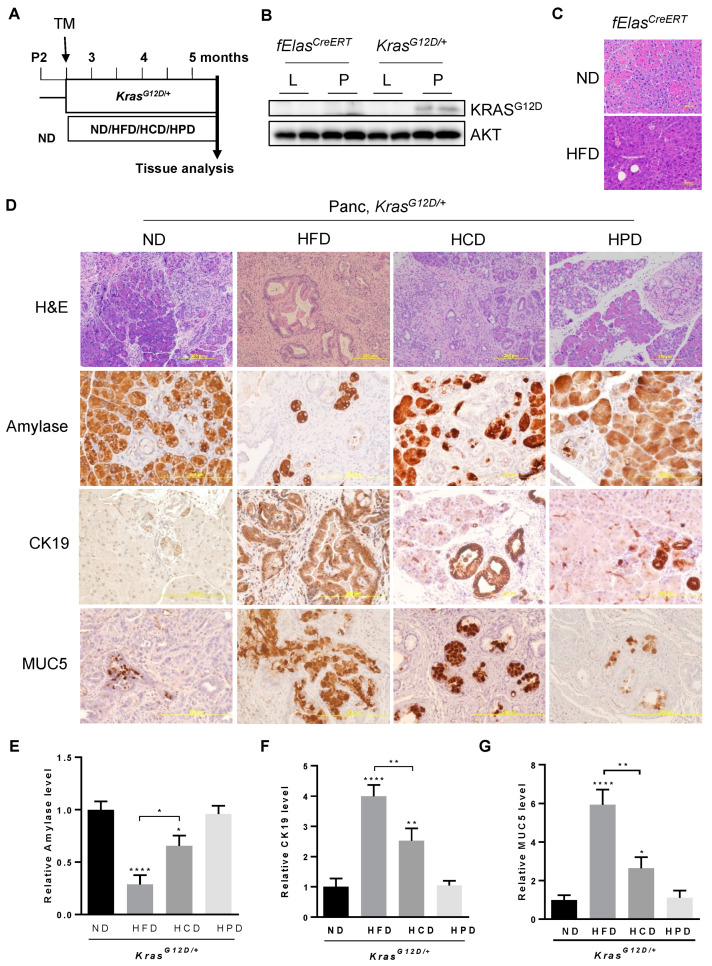
Effects of different macronutrient diets on oncogenic KRAS-mediated pancreatic neoplastic lesions. (**A**) Experimental timeline. *KRAS^G12D/+^* mice were treated with Tamoxifen at the age of about 75 days for 5 days to allow the expression of *KRAS^G12D/+^* specifically in adult acinar cells, separated into four groups fed an ND, HFD, HCD or HPD for 10 weeks, and then euthanized so that pancreatic pathological changes could be analyzed. (**B**) The protein levels of KRAS^G12D^ in the pancreata and livers of *KRAS^G12D/+^* and *fElas^CreERT^* mice. Original image of western blot can be found at Figure S1. (**C**) H&E staining of sections of representative pancreata from *fElas^CreERT^* mice fed an ND and an HFD under the same conditions as described in (**A**). Magnification: 200×. (**D**) H&E and IHC staining of paraffin sections of representative pancreata from *KRAS^G12D/+^* mice under ND, HFD, HCD, or HPD treatment for 10 weeks, using antibodies against amylase, CK19, and Muc5. Magnifications: 100× for H&E and 200× for IHC. (**E**–**G**) Quantification of the above IHC stains with Fiji ImageJ software by measuring the integrated density (IOD) and the areas. *, **, and ****, *p*-value < 0.05, 0.01, and 0.0001, respectively.

To assess the impact of diets enriched in different macronutrients, such as fats, carbohydrates, and proteins, on pancreatic pathogenesis induced by oncogenic KRAS^G12D^, we utilized the previously described *fElas^CreERT^;Kras^LSL-G12D/+^* mouse model (hereafter called *Kras^G12D/+^* after tamoxifen induction) [30,32]. Adult mice at two and a half months old were treated with tamoxifen for 5 days to induce pancreatic acinar cell-specific *Kras^G12D/+^* expression at endogenous levels, followed by feeding with an HFD, an HCD, an HPD, or an ND for 10 weeks (Figure 1A). To confirm that KRAS^G12D^ is specifically expressed in the pancreata of *Kras^G12D/+^* mice, we extracted proteins from the pancreas and liver of *Kras^G12D/+^* mice and *fElas^CreERT^* control mice. Our data showed that KRAS^G12D^ was expressed in the pancreata of the *KRAS^G12D^* mice, but not in the livers of the *Kras^G12D/+^* mice or the pancreata and livers of the *fElas^CreERT^* control mice (Figure 1B). Previous studies showed that HFD can synergize with oncogenic KRAS^G12D^ to promote PDAC development [12]. To test the effect of HFD in mice in the absence of oncogenic KRAS, we fed *fElas^CreERT^* mice with either an ND or an HFD under the same conditions as *Kras^G12D/+^* mice. Our data showed that the pancreata of the HFD-fed fElas^CreERT^ mice are histologically normal without any signs of ADM, inflammation, or PanIN lesions compared to those fed an ND, suggesting that the HFD has a negligible impact on the pancreas in the absence of oncogenic KRAS (Figure 1C). By contrast, histopathological analyses of H&E stained pancreatic tissue sections of the HFD-fed *KRAS^G12D/+^* mice revealed widespread lesions characterized by drastically diminished acinar cell clusters that were replaced by extensive inflammation and ductal structure (Figure 1D, H&E). Although less severe than the HFD-fed mice, the pancreata of the HCD-fed *Kras^G12D/+^* mice also exhibited substantially reduced acinar clusters and enhanced ductal metaplasia compared to those of the ND-fed *Kras^G12D/+^* mice, indicating that an HCD promotes neoplastic lesions in cooperation with KRAS^G12D^ in the pancreas. By contrast, the HPD did not significantly alter pancreatic pathologies induced by KRAS^G12D^ compared to the ND, indicating differential effects of different macronutrient contents in diets on pancreatic pathogenesis in association with oncogenic KRAS.

Next, we analyzed the changes in pancreatic acinar cells, ductal metaplasia, and PanIN lesions using biomarkers by IHC. Pancreatic amylase, a specific marker for functional pancreatic acinar cells, decreased dramatically by about 70% in the pancreata of *Kras^G12D/+^* mice fed an HFD, which is consistent with previous observations that the HFD facilitated pancreatic ADM, a precursor for PanIN lesions and PDAC development [12,30]. HCD treatment reduced amylase content by about 35% compared to the ND, albeit with a lower severity than that of the HFD (Figure 1D,E). By contrast, chronic consumption of HPD had little effect on amylase content, which was indistinguishable from that of the ND control. These data suggest that in contrast to the negligible effects similarly exerted by both the HPD and the ND, the HCD significantly reduced amylase content albeit less effectively than the HFD.

An abnormal increase in the expression of cytokeratin 19 (CK 19), a ductal epithelial marker, is often observed in the development of pancreatic neoplasia. Consistent with previous observations that chronic consumption of an HFD exacerbated KRAS-mediated development of irreversible ADM, rapid PanIN lesions, and invasive PDAC [12,26,30], we observed dramatic increases in CK19 levels and abnormally abundant ductal structures throughout the pancreata of the HFD-fed *Kras^G12D/+^* mice (Figure 1D,F). To a lesser but still significant extent, chronic consumption of an HCD elevated CK19 levels in the pancreata compared to that of the ND-fed *Kras^G12D/+^* mice, indicating that chronic HCD also promotes the development of pancreatic neoplastic transformation. By contrast, chronic HPD had quantitatively insignificant effects on CK19 levels compared to the ND in *Kras^G12D/+^* mice, suggesting that chronic high protein intake does not impose significant adverse effects on mutant KRAS-mediated pancreatic neoplastic development.

Furthermore, we analyzed the changes in Mucin 5 (MUC5), which is not expressed in the normal pancreas but gains expression ectopically in the early stage of about 70% of PanIN cases and 85% of PDAC [33]. Consistent with the reported enhancement of MUC5 expression in PanIN lesions and PDAC, obesogenic HFD markedly increased MUC5 levels across the *Kras^G12D/+^*-harboring pancreata examined (Figure 1D–G). Similarly, chronic HCD significantly increased MUC5 expression in *Kras^G12D/+^*-expressing pancreata albeit to a lesser extent than HFD. By contrast, HPD exerted negligible effects on MUC5 levels in KRAS^G12D/+^-expressing pancreata, which was similar to the ND. Taken together, all the above data indicate that different macronutrients have differential effects on promoting KRAS-mediated pancreatic neoplastic lesions.

### 3.2. HCD, but Not HPD, Instigates Inflammation and Fibrosis in Kras^G12D/+^-Expressing Pancreata

**Figure 2:**
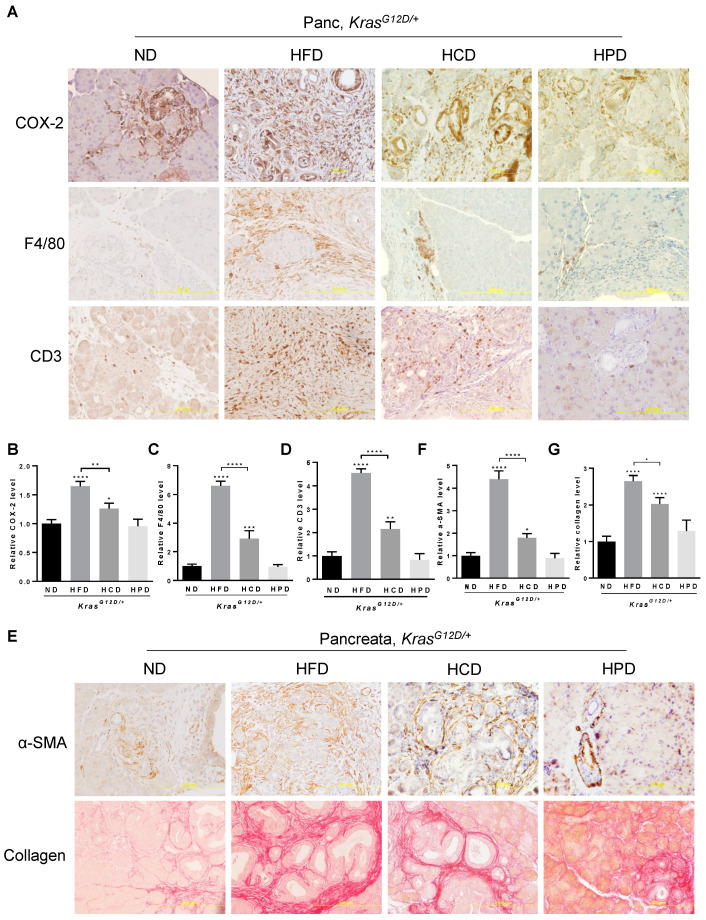
Effects of different macronutrient diets on oncogenic KRAS-mediated pancreatic inflammation and fibrosis in mice. (**A**) IHC staining of the pancreas from the Kras^G12D/+^ mice after 10-week ND, HFD, HCD, or HPD treatment with antibodies against COX-2, F4/80, or CD3. Magnification: 200×. (**B**–**D**) Quantification of each indicated staining in (**A**) with Fiji ImageJ software by measuring the integrated density (IOD) and area. (**E**) IHC staining of α-SMA (upper panel) and Sirius Red staining of collagens (lower panel) in pancreatic sections from Kras^G12D/+^ mice after 10-week ND, HFD, HCD, or HPD treatment. Magnification: 200×. (**F**,**G**) Quantification of each indicated staining in (**E**) with Fiji ImageJ software by measuring the integrated density (IOD) and area. *, **, *** and ****, *p*-value < 0.05, 0.01, 0.001 and 0.0001, respectively.

Chronic consumption of HFD is known to promote metabolic abnormalities leading to overweight and obesity with meta-inflammation and inflammatory tissue damage, which are associated with the initiation and development of PanIN and PDAC [12,30]. Consistent with previous reports, HFD significantly enhanced the levels of COX-2, a common marker of tissue inflammation, in the pancreata of *Kras^G12D/+^* mice. Chronic HCD increased COX-2 levels by about 80% of that caused by the HFD (Figure 2A,B). In contrast to both HFD and HCD, chronic HPD had little effects on COX-2 levels, which were comparable to that of the ND in *Kras^G12D/+^* mice, suggesting that diets rich in proteins have no detectable adverse effects on pancreatic inflammation of *Kras^G12D/+^* mice.

The occurrence and intensity of pancreatic inflammation are associated with the levels of infiltration of a variety of immune cells. Analyses of macrophage marker F4/80 using IHC revealed that HFD considerably increased the levels of F4/80 to about 6-fold that of the ND control (Figure 2A–C), indicating significant infiltration of macrophages to the pancreata upon chronic HFD intake in the *Kras^G12D/+^* mice. Chronic HCD enhanced F4/80 about 2-fold compared to ND, indicating that HCD also enhances the infiltration of macrophages to the pancreata albeit with a lesser potency than the HFD. On the other hand, chronic HPD remained the same as the ND in promoting the infiltration of macrophages into the *Kras^G12D/+^*-bearing pancreata. Similarly, we found that CD3^+^ T cell infiltration to the pancreata was remarkably increased 4-fold under HFD and 2-fold HCD compared to the ND controls (Figure 2A–D). By contrast, chronic HPD had insignificant effects on CD3 levels, indicating that HPD lacks the ability to promote T cell infiltration into the KRAS^G12D^-expressing pancreata.

Inflammation is known to cause abnormal activation of stellate cells and subsequent secretion and deposition of extracellular matrix (ECM) proteins that contribute to tissue fibrosis, a process associated with PDAC development. We found that HCD increased the levels of α-SMA and Sirius Red staining of collagen, markers of myofibroblastic stromal cell activation and collagen deposition, respectively, in the pancreata of *Kras^G12D/+^* mice (Figure 2E–G), although such increases were about 55% and 65% of that caused by HFD, respectively, indicating that HFD remains more potent than HCD in promoting pancreatic fibrosis in alliance with oncogenic KRAS. By contrast, HPD appeared to only slightly increase the intensity of α-SMA and Sirius Red staining at limited foci; however, the difference did not reach a statistical significance compared to ND (Figure 2E–G), suggesting that chronic high protein intake has little effects on pancreatic fibrosis.

### 3.3. Chronic HCD, but Not HPD, Accelerates Cell Proliferation and Decreases the Survival of KRAS^G12D/+^ Mice

**Figure 3:**
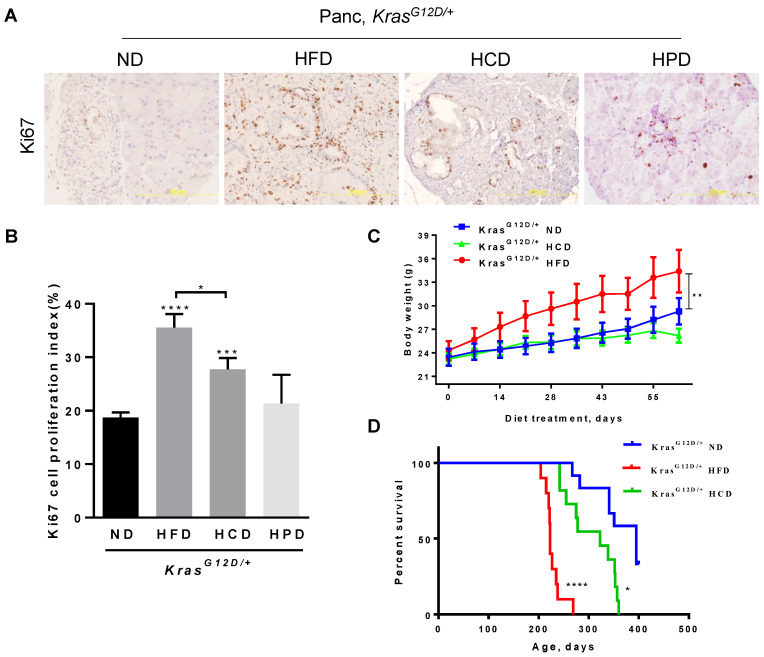
Differential effects of different macronutrient diets on pancreatic cell proliferation, body weights, and survival rates in KRAS^G12D/+^ mice. (**A**) Ki67 IHC staining was performed to detect pancreatic cell proliferation of Kras^G12D/+^ mice after 10-week ND, HFD, HCD, or HPD treatment, with hematoxylin counterstaining. Magnification: 200×. (**B**) Quantification of pancreatic cell proliferation by manually counting Ki67-positive nuclei of ductal cells. (**C**) Body weight of Kras^G12D/+^ mice fed an ND, an HFD, or an HCD. At 75 days old, tamoxifen was administrated to mice by intraperitoneal injection for 5 days to activate Kras^G12D/+^ expression. ND, HFD or HCD treatment started on day 80 for 10 weeks. Body weight was measured weekly. (**D**) Survival rates of *Kras^G12D/+^* mice treated with HFD (red line), HCD (green line), or ND (blue line). *, **, *** and ****, *p*-value < 0.05, 0.01, 0.001 and 0.0001, respectively.

Oncogenic KRAS is a master regulator of cell proliferation required for neoplastic progression, which is often further accelerated by several risk factors. IHC analyses of Ki67, a marker of cell proliferation and cancer diagnosis, revealed that chronic consumption of an HFD remarkably increased Ki67 levels in the pancreata of *Kras^G12D/+^* mice. The HCD also significantly elevated Ki67 levels albeit at about 60% of the potency of the HFD (Figure 3A,B). In comparison, the HPD exerted insignificant effects on the levels of Ki67, which were comparable to that of *Kras^G12D/+^* pancreata under an ND. These data indicate that both the HFD and the HCD, but not the HPD, are effective in promoting mutant KRAS-induced aberrant pancreatic ductal cell proliferation.

Although it did so with a lesser potency than the HFD, the HCD significantly accelerated several specific parameters characteristic of PDAC development, which are in marked contrast to the ineffectiveness of the HPD or the ND in the pancreata of *KRAS^G12D/+^* mice. To further understand the sequelae of these changes on the mice, we analyzed the corresponding changes in body weight and survival. Our results showed that HFD significantly increased the body weight and, correspondingly, significantly reduced the survival rate of *Kras^G12D/+^* mice, consistent with the known role of obesity as a major risk factor for pancreatic cancer. In contrast to the HFD, the HCD was ineffective in increasing the body weight but was still significant in reducing the survival rate, even though at a lesser potency than the HFD (Figure 3C,D). The HPD exerted none of the specific alterations characteristic of pancreatic lesions and PDAC development compared to the ND; thus, we did not evaluate its effect on the survival rate of HPD-fed *Kras^G12D/+^* mice.

### 3.4. COX-2 Mediates the Effects of HCD and KRAS^G12D^ on Promoting Pancreatic Neoplasia

**Figure 4:**
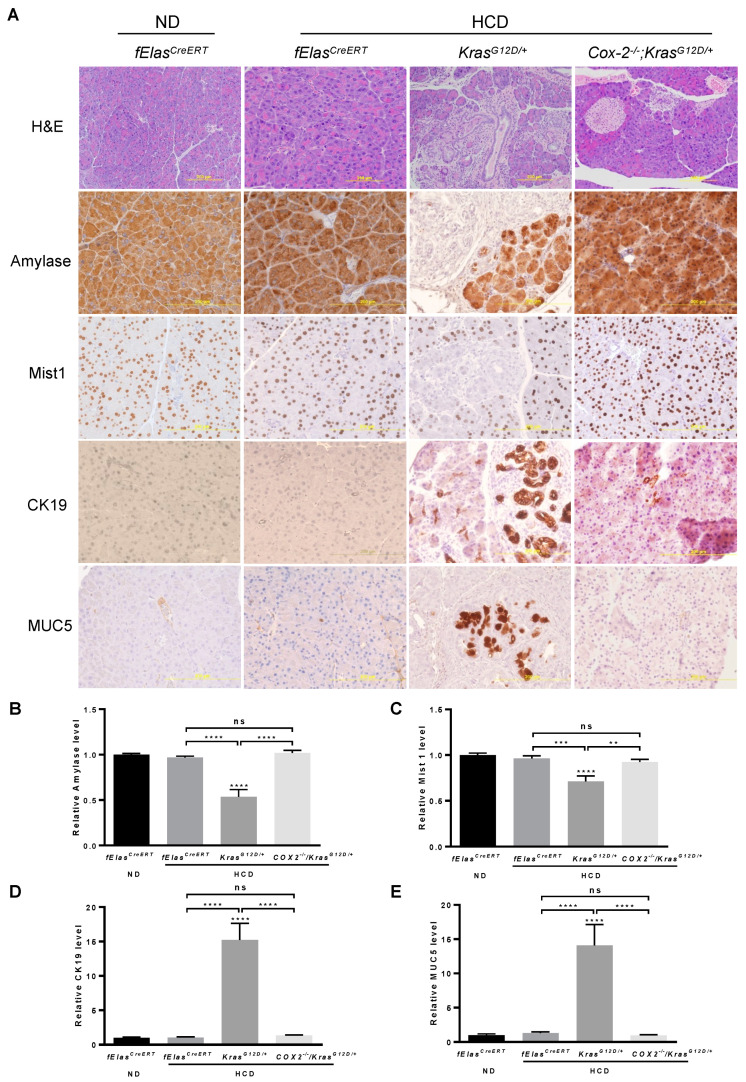
Effects of COX-2 deficiency on HCD and KRAS^G12D/+^ mediated pancreatic neoplastic development. (**A**) The ablation of COX-2 in HCD-fed *Kras^G12D/+^* mice preserved pancreatic acinar cells, inhibited ductal-like cell formation, and reduced PanIN lesions. Seventy-five-day old male and female mice were induced by tamoxifen for the indicated gene expression or deletion in pancreatic acinar cells and subsequently treated with HCD or ND for 10 weeks. Paraffin sections of the pancreata from these mice were analyzed by H&E or IHC with antibodies against amylase, Mist1, CK19, or Muc5 (200× magnification). (**B**–**E**) Quantification of the stains in (**A**) with Fiji ImageJ software. NS, not significant. **, *** and ****, *p*-value < 0.01, 0.001 and 0.0001, respectively.

Inflammation or pancreatitis was reported to synergize with oncogenic KRAS to promote pancreatic tumorigenesis even in insulin-expressing endocrine cells that are highly refractory to transformation, suggesting that diet-induced inflammation may play a key role in oncogenic KRAS-mediated pancreatic tumorigenesis [11]. It has been shown that chronic HFD significantly elevated COX-2 levels, leading to the development of extensive inflammation and neoplasm in the *Kras^G12D/+^* pancreas, while pancreatic acinar cell-specific ablation of *Cox-2* abolished the effects of HFD on stimulating oncogenic KRAS-induced inflammation, PanIN lesions, and PDAC [12,32]. To understand whether inflammation is critical for the cooperative effects of chronic HCD and KRAS^G12D^ on promoting pancreatic neoplastic lesions and PDAC development, we fed the age-matched male and female (80-day old) *Kras^G12D/+^* or *Kras^G12D/+^;Cox-2^−/−^* mice with an HCD for 10 weeks after tamoxifen induction. *fElas^CreERT^* mice fed an ND or an HCD were used as controls. By analyzing H&E stained sections, we found that pancreatic acinar cell depletion of *Cox-2* markedly reversed pancreatic neoplastic morphologies including inflammation, stromal fibrosis, and PanIN lesions induced cooperatively by HCD and KRAS^G12D^ (Figure 4A). IHC analyses of amylase and Mist1, markers of functional pancreatic acinar cells, showed that the depletion of pancreatic acinar cell COX-2 substantially rescued the loss of amylase and Mist1 induced by HCD and KRAS^G12D^, leading to levels resembling that of the ND- or HCD-fed *fElas^CreERT^* control mice (Figure 4A–C). Similarly, COX-2 deficiency remarkably inhibited pathogenic ductal cell formation and PanIN lesions as indicated by the reduction of CK19+ and MUC5+ stains induced by HCD and oncogenic KRAS^G12D^ (Figure 4A,D,E). These results suggest that pancreatic acinar cell COX-2 mediates a spectrum of pancreatic lesions induced cooperatively by HCD and oncogenic KRAS.

### 3.5. COX-2 Mediates the Effects of High Carbohydrate Diets and Oncogenic KRAS on Promoting Pancreatic Inflammation and Fibrosis

**Figure 5:**
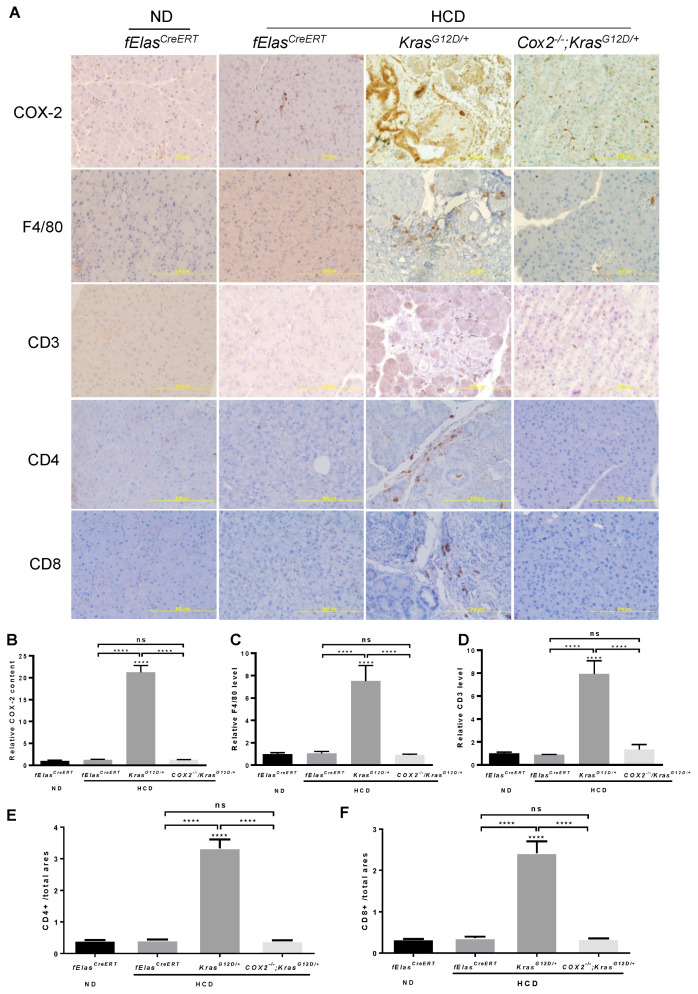
Effects of COX-2 deficiency on the HCD and KRAS^G12D/+^ mediated pancreatic inflammation and immune cell infiltration. (**A**). Immunohistochemical analyses of mouse pancreata from the indicated four groups with antibodies against COX-2, F4/80, CD3, CD4, and CD8. Magnification: 200×. (**B**–**F**). Quantification of IHC stains of COX-2, F4/80, and CD3, CD4, and CD8, respectively. NS, not significant. ****, *p*-value < 0.0001.

To understand whether the effects of a chronic HCD on promoting oncogenic KRAS-mediated ADM, PanIN lesions, and PDAC involve inflammation, we analyzed the changes in markers for inflammation and immune cell infiltration in the *KRAS^G12D+^*-expressing pancreas. As expected, the induction of COX-2 by HCD in *KRAS^G12D/+^* pancreata was completely abolished when *Cox-2* gene was selectively deleted in pancreatic acinar cells (Figure 5A,B). Correspondingly, we found that the increases in macrophage-specific marker F4/80 by chronic HCD and KRAS^G12D^ were reversed to the basal levels as that in the ND- or HCD-fed *fElas^CreERT^* mice after ablating acinar cell *Cox-2*, indicating significant inhibition of the recruitment of F4/80^+^ cells (Figure 5A–C). The significant increases in the levels of CD3, CD4 and CD8, markers for all T cells, T helper cells and cytotoxic T cells, respectively, by HCD and KRAS^G12D^ were also reversed upon the loss of COX-2, indicating the significant inhibition of T cell infiltration, a sign of attenuation of the aggravated immune reaction as well (Figure 5A,D–F). These results indicate that inflammation or the heightened immune reaction in the damaged pancreata likely mediates the detrimental effects that resulted from the cooperative interaction between the HCD and KRAS^G12D^.

**Figure 6:**
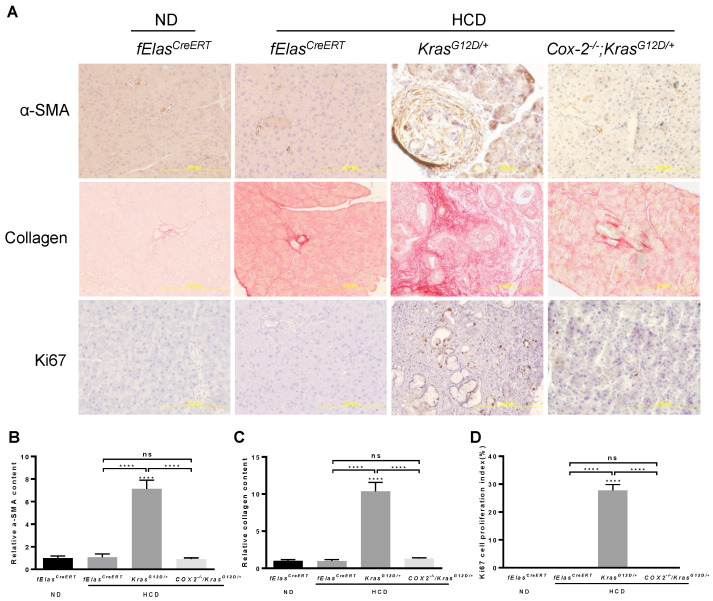
Effects of COX-2 deficiency on the HCD and KRAS^G12D/+^ mediated pancreatic fibrosis and cell proliferation. (**A**) IHC analyses of mouse pancreata from the indicated four groups with antibodies against α-SMA and Ki67, as well as Sirius Red staining for collagen deposition. Magnification: 200×. (**B**,**C**) Quantification of α-SMA and Sirius Red stains with Fiji ImageJ software. (**D**) Quantification of Ki67 staining by manually counting positively stained nuclei of ductal cells. NS, not significant. ****, *p*-value < 0.0001.

Abnormal and persistent inflammation or immune reaction is known to cause tissue damage and fibrosis. In agreement with the reversal of an aggravated inflammation, depletion of COX-2 abolished the synergistic interaction between chronic HCD and KRAS^G12D^ in mediating the activation of stellate cells as marked by the substantial reduction in α-SMA, as well as fibrosis as marked by Sirius Red staining for collagen (Figure 6A–C), indicating that pancreatic COX-2 plays an important role in mediating the development of fibrosis induced by chronic HCD and KRAS^G12D^. Furthermore, ablation of COX-2 resulted in a significant reduction in abnormal ductal cell proliferation induced by HCD and oncogenic KRAS as evaluated by IHC staining of Ki67 (Figure 6A,D).

## 4. Discussion

Mutant KRAS alone is insufficient to drive full-blown PDAC and pancreatic cancer risk factors as a second hit are required. Increased aerobic glycolysis is a hallmark of cancer, for which a carbohydrate-rich diet is considered a more direct source to fuel cancer cell proliferation compared to an HFD and an HPD. On the other hand, obesity is a known modifiable risk factor for pancreatic cancer and chronic consumption of obesogenic HFD was shown to exacerbate PDAC development. However, it is not clear how these different dietary macronutrients impact pancreatic tumorigenesis. A better understanding of the differential effects of dietary macronutrients in PDAC development will provide new insights into development of possible effective nutraceutical intervention strategies. In this study, we have made several important observations regarding the differential effects of HCD, HFD, and HPD in oncogenic KRAS-mediated pancreatic tumorigenesis. First, we found that the chronic HFD exhibited a more severe effect on transforming acinar cells than the HCD, as judged by changes in the histopathological features and a series of molecular markers of ADM, inflammation, fibrosis, PanIN lesions, and host survival. Second, the chronic HPD exerted negligible adverse effects on PDAC development compared to the chronic ND in the KRAS^G12D^ mice. Third, albeit with a lesser potency than HFD, the chronic HCD significantly reduced the survival rate of the KRAS^G12D^-expressing mice compared to the ND. Finally, pancreatic inflammation and associated pathologies induced by the synergistic cooperation between HCD and oncogenic KRAS can be effectively blocked by the ablation of acinar cell COX-2, suggesting that pancreatic acinar cell inflammation plays a critical role in mediating the adverse effects of HCD on PDAC development.

The synergistic effects of HFD and oncogenic KRAS on promoting pancreatic neoplasia appeared to be more conspicuous than that of HCD and oncogenic KRAS. This observation is concordant with our previous results, showing that chronic HFD promoted oncogenic KRAS-mediated aerobic glycolysis more effectively than chronic HCD even though HCD is considered as a more direct source of glucose for glycolysis [32]. The mechanism underlying such a preference is still not well understood. One possibility is that chronic HFD induces more extensive inflammation from the oxidized lipids-mediated lipotoxicity, leading to a more intense hyperactivation of oncogenic KRAS to drive aerobic glycolysis. Understanding why HFD and HCD differentially drive inflammation and fuel glycolysis may thus provide important insights into effective intervention strategies for this highly lethal disease. By contrast, a protein-rich diet showed little effects on oncogenic KRAS-mediated pancreatic neoplasm, suggesting that consuming a protein-rich diet could be a more viable dietary intervention approach for pancreatic cancer patients.

Although it was not as pronounced as with the HFD, the HCD enhanced pancreatic inflammation, fibrosis, ADM, PanIN lesions, and proliferation in the pancreata of the KRAS^G12D^ mice. Ablation of COX-2 in pancreatic acinar cells substantially alleviated inflammation and neoplastic lesions, indicating that pancreatic inflammation plays an important role in mediating the cooperative effects of the HCD and oncogenic KRAS on PDAC development. Previous studies showed that ablation of pancreatic acinar cell *Cox-2* significantly reduced inflammation, fibrosis, and PanIN lesions in oncogenic KRAS mice fed an HFD. Consistently, treatment with Celecoxib, a selective COX-2 inhibitor, resulted in similar effects [12,32]. COX-2 is a critical enzyme for the synthesis of the pro-inflammatory lipid mediator prostaglandin (PGE2) from membrane arachidonic acid, which is secreted to neighboring cells or circulation to act on the PGE2 receptors EP1-4, members of GPCR family expressed on macrophages and other immune cells, leading to tissue-wide inflammation, immune cell infiltration, and fibrosis that together contribute to PDAC progression. Our data together suggest that diet-induced inflammation plays a prominent role in inducing pancreatic neoplasm and is essential for licensing the adverse tumorigenic effect of oncogenic KRAS on pancreatic acinar cells. Therefore, selective nutraceutical diets or drugs with anti-inflammatory effects may hold a great promise in preventing pancreatic cancer development.

It is still not clear why the chronic HCD showed significant adverse effects compared to the ND, including the reduction in the survival rate of KRAS^G12D^ mice. It is noteworthy that the major component of the carbohydrates in the HCD is sucrose, a disaccharide made of glucose and fructose; however, the major component of the carbohydrates in the ND is starch, a polymeric carbohydrate. Several cohort studies suggest that patients ingesting high sugar-sweetened beverages or free fructose are at greater risks of developing pancreatic cancer [34,35]. Fructose was reported to induce transketolase flux to promote pancreatic cancer growth independent of glucose [36]. Dietary fructose improves intestinal cell survival and nutrient absorption and studies have shown that daily oral administration of high-fructose corn syrup in adenomatous polyposis coli (APC) mutant mice led to a substantial increase in tumor size and tumor grade in the absence of obesity. Within the tumors, fructose was converted to fructose-1-phosphate, leading to activation of glycolysis and increased synthesis of fatty acids that support tumor growth [37,38]. More experiments are needed to address if sucrose disaccharides and starch polysaccharide act differently in the context of oncogenic KRAS.

## 5. Conclusions

In summary, our study reveals that different macronutrients have differential effects on oncogenic KRAS-mediated pancreatic neoplasia. As with the chronic HFD, the HCD cooperates with oncogenic KRAS to promote pancreatic inflammation, fibrosis, PanIN lesions, and PDAC development albeit with a lesser potency. Targeted inhibition of diet-induced pancreatic inflammation alleviates pancreatic neoplasm. Our study suggests that dietary macronutrients differentially impact pancreatic cancer susceptibility and HPD may represent the desired, favorable dietary choice compared to HFD and HCD for pancreatic cancer patients.

## Data Availability

The data presented in this study are available on request from the corresponding author.

## Supplementary Materials

The following are available online at https://www.mdpi.com/article/10.3390/cancers14112723/s1, Figure S1: Whole western blot and densitometry readings/intensity ratio of mutant KRAS protein level in liver and pancreas of *fElas^CreERT^* and *Kras^G12D/+^* mice treated with Normal Diet.
