# Supplementary material for: Differential Effects of Dietary Macronutrients on the Development of Oncogenic KRAS-Mediated Pancreatic Ductal Adenocarcinoma
Source: Cancers (Basel). 2022 May 31;14(11):2723. doi: 10.3390/cancers14112723 (PMC9179355; doi:10.3390/cancers14112723)
Supplement: Supplementary file 1 [file cancers-14-02723-s001.zip › cancers-1742030-supplementary.docx]

L P L P

*fElas^CreER^ Kras^G12D/+^*

|  | *fElas^CreERT^* | | | | *Kras^G12D/+^* | | | |
| --- | --- | --- | --- | --- | --- | --- | --- | --- |
|  | L | | P | | L | | P | |
| KrasG12D/AKT | 0.000 | 0.000 | 0.000 | 0.000 | 0.000 | 0.000 | 0.072 | 0.100 |


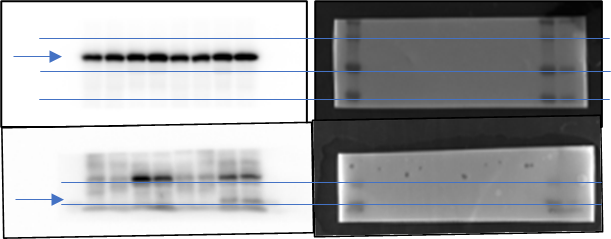


Kras^G12D^

21KD

75KD

50KD

37KD

AKT

60KD

25KD

20KD

**Figure S1.** Whole western blot and densitometry readings/intensity ratio of mutant KRAS protein level in liver and pancreas of *fElas^CreERT^* and *Kras^G12D/+^* mice treated with Normal Diet.

L: Liver; P: Pancreas.
